# NAOMI: The trials and tribulations of implementing a heroin assisted treatment study in North America

**DOI:** 10.1186/1477-7517-6-2

**Published:** 2009-01-21

**Authors:** Candice C Gartry, Eugenia Oviedo-Joekes, Nancy Laliberté, Martin T Schechter

**Affiliations:** 1 CIHR Canadian HIV Trials Network and the Centre for Health Evaluation & Outcome Sciences (CHEOS), 620B – 1081 Burrard Street, Vancouver, BC, V6Z 1Y6, Canada; 2 Centre for Health Evaluation & Outcome Sciences (CHEOS), 620B – 1081 Burrard Street, Vancouver, BC, V6Z 1Y6, Canada; 3 UBC School of Population and Public Health, 5804 Fairview Ave, Vancouver, BC, V6T 1Z3, Canada

## Abstract

**Background:**

Opioid addiction is a chronic, relapsing disease and remains a major public health challenge. Despite important expansions of access to conventional treatments, there are still significant proportions of affected individuals who remain outside the reach of the current treatment system and who contribute disproportionately to health care and criminal justice costs as well as to public disorder associated with drug addiction.

The NAOMI study is a Phase III randomized clinical trial comparing injectable heroin maintenance to oral methadone. The study has ethics board approval at its Montréal and Vancouver sites, as well as from the University of Toronto, the New York Academy of Medicine and Johns Hopkins University.

The main objective of the NAOMI Study is to determine whether the closely supervised provision of injectable, pharmaceutical-grade opioid agonist is more effective than methadone alone in recruiting, retaining, and benefiting chronic, opioid-dependent, injection drug users who are resistant to current standard treatment options.

**Methods:**

The case study submitted chronicles the challenges of getting a heroin assisted treatment trial up and running in North America. It describes: a brief background on opioid addiction; current standard therapies for opioid addiction; why there is/was a need for a heroin assisted treatment trial; a description of heroin assisted treatment; the beginnings of creating the NAOMI study in North America; what is the NAOMI study; the science and politics of the NAOMI study; getting NAOMI started in Canada; various requirements and restrictions in getting the study up and running; recruitment into the study; working with the media; a status report on the study; and a brief conclusion from the authors' perspectives.

**Results and conclusion:**

As this is a case study, there are no specific results or main findings listed. The case study focuses on: the background of the study; what it took to get the study started in Canada; the unique requirements and conditions of getting a site, and the study, approved; working with the media; recruitment into the study; a brief status report on the study; and a brief conclusion from the authors' perspectives.

**Trail Registration:**

ClinicalTrials.gov registration number: NCT00175357

## Background

Opioid addiction is a chronic, relapsing disease and remains a major public health challenge in North America with an estimated 80,000 opioid users in Canada alone [1]. It is estimated that there are 20,000 opioid dependent persons in Vancouver and at least 6,000 in Montréal, these representing only two of Canada's larger cities [1]. Despite important expansions of access to conventional treatments, there are still significant proportions of affected individuals, many with multiple co-morbidities, who remain outside the reach of the current treatment system and who contribute disproportionately to health care and criminal justice costs as well as to public disorder associated with drug addiction [2].

Untreated opioid addiction can lead to overdose, infectious diseases, loss of regular social and economic functioning, and extensive engagement in both drug-related and drug acquisition crime [3-6]. Opioid addiction is also highly correlated with mental illness and the combination of these two is a predictor of poorer treatment outcomes [7-9]. At a societal level, there are associated costs to public health and health care as well as to the welfare and criminal justice systems in dealing with these risk and harm phenomena. In Canada, illicit drug use costs in 2002 were estimated at $40 billion or $1,267 per capita with the three highest contributors being loss of productivity, direct health care and law enforcement [10]. In 1996, Vancouver's Medical Health Officer reported that the increase in injection drug use seen in Vancouver was resulting in an increased incidence of HIV/AIDS and Hepatitis, increased hospital and emergency service utilization for treatment of HIV-related diseases, septicaemia and endocarditis, as well as increased ambulance responses and emergency room visits related to drug overdoses. In addition, the report noted a related increase in fetal exposure to addictive substances, increased pressure on community-level outreach nursing and medical services, and an increased need for community-level hospice palliative care [11].

Methadone maintenance therapy (MMT) has been the primary substitution treatment for opioid addiction in North America since the 1960's [12,13]. MMT efficacy has been largely proved with respect to various outcomes [14-17] – it decreases the cravings, decreases the number of injections, and can sometimes lead to abstinence. However, like other treatments for diseases such as cancer or HIV, MMT is not effective for everyone. Some patients on MMT are not retained long enough to benefit from the program or show limited response, continuing to use illicit drugs outside of the treatment setting [18]. Several factors have been highlighted as predictors of lack of efficacy of MMT or barriers to access (and retention) in the program [19-25]. These include: inadequate methadone doses; user fees; punitive urine testing (e.g. complete abstinence from any drug use required for continued MMT); and lack of a 'high' associated with MMT. While many of these factors can be improved upon, even when MMT is optimally delivered (i.e. high dosage, psychosocial support, treatment of co-morbidities), there remains a sub-sample of between 15% and 25% of patients who do not benefit from this treatment [26,27]. For example, the MMT group in the German Heroin Assisted Therapy (HAT) trial received an optimized version of MMT (compared to what is available in their communities) and still 30% and 50% were considered 'non-responders' with respect to their health and illicit drug use scores respectively [28]. As such, MMT is effective but can be sub-optimal and it is not always successful.

Buprenorphine, particularly suboxone (a combination of buprenorphine and naloxone) – a partial agonist, has been approved in the United States, and recently in Canada, for substitution therapy. While there is limited data on the effectiveness of this treatment in addiction therapy, use of suboxone has shown that it may only be truly effective for those who use low doses of opioids or who are on low doses of methadone. In fact, a recent review of 13 clinical trials, 12 of which were double blind, comparing buprenorphine maintenance with either placebo or MMT for opioid dependence concluded that buprenorphine appeared to be significantly less effective than methadone in retaining patients in treatment [29].

### Why the need for a HAT trial?

The first question that can be asked is 'Is there really a need for a trial of heroin assisted therapy?' The need for such a trial was noted in the Le Dain Commission's Report in 1972 [30]. This report was produced by the Canadian Government Commission on Inquiry into the Non-Medical Use of Drugs. Included in this report is a recommendation for the "implementation of a heroin prescription trial for addicts who could not be attracted into conventional forms of opioid addiction treatment".

In the United Kingdom (UK) heroin prescription has been part of the addiction treatment system since 1926 [30-32]. In the nineties, Switzerland opened heroin assisted treatment (HAT) clinics in response to the public health problem caused by the use of illicit heroin [33,34]. The Swiss experience showed that heroin prescription delivered under supervision in clinics was both safe and feasible [33,34]. Moreover, these patients improved their health, reduced their use of illicit drugs and illegal income, and also committed fewer drug and property related offences [35,36]. These improvements were sustained over time, even after leaving the treatment [37]. In 1999 a World Health Organization's expert panel issued a report in which they recognized this success; however, the panel also felt that it was not possible to determine whether these improvements were due to HAT or the ancillary psychosocial services that the participants received [38].

After the success of the Swiss model, several European countries followed their initiative. The Netherlands, Germany and Spain, followed later by Canada and the UK, implemented randomized controlled HAT trials in order to better answer the question of the effectiveness of supervised HAT in their contexts [26-28,39-41]. In addition, in 2008 Denmark announced the approval of HAT as part of their addiction treatment program and Belgium started a Randomized Controlled Trial (RCT) comparing injected and inhaled heroin (Dutch model [26]) versus oral methadone. The Dutch, German and Spanish trials results showed that HAT was more effective than oral MMT in the areas of improved health, psychosocial adjustment and illicit drug use among long-term, socially excluded heroin-dependent people who were not benefiting from the available treatments [26-28,40].

In Switzerland and the Netherlands, HAT is now part of the addiction treatment system, and Germany is in the process of requesting registration for diamorphine (DAM). However, in Spain the government denied the request of HAT for opioid-dependence treatment and currently only allows it under compassionate use.

Although evidence is building that HAT is an effective treatment alternative for opioid dependence, more work is still to be done. Even with the study results on HAT currently published, many countries still do not support the use of heroin as a treatment option for opioid dependence largely due to the stigma associated with this drug, as well as the politics associated with treating those dependent on heroin with heroin. Some feel that while HAT worked in some countries, it may not work in their own because of different cultural and societal issues.

While Denmark approved HAT without a clinical trial, Belgium researchers have been trying for some time to get HAT approved and, at this point, only have approval for a RCT using MMT as an active comparator. In Switzerland, the provision of HAT was approved after a referendum was passed and the German trial went ahead only after a change in their government. In Spain, the debate went on for years before a RCT of HAT was approved. Thus, despite the recommendations made by the Le Dain Commission so many years ago, the successful experiences in other countries, and that addiction treatment policies, like any medical or public health practice, should be evidence based – and evidence does exists [42], the only hope of possibly having DAM registered as a treatment for opioid addiction in Canada meant that a RCT of HAT was needed to examine whether HAT would be effective in the Canadian context.

### Understanding HAT

Heroin (injected) assisted treatment is aimed at a very select group of opioid users. The target population for HAT have been using heroin (or other illicit opiates) for several years, have severe health and psychosocial problems associated with their drug use, and have not benefited currently or in the past from available therapies such as MMT. HAT should not be considered as a replacement for MMT or buprenorphine, but rather as an additional treatment option for a specific sub-population and for whom MMT and/or buprenorphine have not been effective. Supervised HAT clinics require daily visits (up to 3 per day) and participants are subject to close monitoring and evaluation while in the clinic. Medically prescribed heroin, while provided at no cost to participants, is not 'free heroin' as it is a tightly controlled and very demanding treatment [43,44].

If one considers the Swiss HAT treatment model as a reference, with 23 clinics working at 91% capacity, HAT accounts for 8% of the substitution treatment [45] being provided in that country. Thus, HAT is clearly an alternative for the most vulnerable and severely affected population of opioid dependent people, who, without HAT treatment, would likely remain outside of the treatment system. The amount of attention and controversy around this treatment, given this important but circumscribed role within the realm of addiction treatment, is to say the least, disproportionate [46].

### NAOMI – the early days

In September 1998 the first North American Opiate Medication Initiative (NAOMI) Working Group was formed with treatment experts, research scientists and bio-ethicists from both the US and Canada. The mandate of the group was to examine HAT as a treatment modality for chronic opiate-dependence and, if thought feasible, develop a scientifically rigorous and ethically defensible study proposal.

The NAOMI Working Group held a series of protocol design meetings from 1998 to 2000 during which time a six-centre RCT proposal was developed. Three sites were to be established in Canada (Vancouver, Toronto and Montréal) with the other three to be established in the US. In 1999 an external international review panel was commissioned by the investigators to review the proposal while at the same time discussions were being held to identify possible sites and funding in the US. As time went on, it became increasingly clear that no US sites would be able to participate nor could any US funding be identified. As a result, the Canadian investigators decided to apply as a Canadian three-site study to the Canadian Institutes of Health Research (CIHR), and the NAOMI study was officially born.

### NAOMI – what is it?

The NAOMI study is a Phase III RCT comparing injectable opioid agonist maintenance (primarily with heroin but also with hydromorphone) to oral methadone. The study is funded by CIHR, Canada's premier scientific research funding body, and has ethics board approval at its Montréal (Université de Montréal) and Vancouver (University of British Columbia and Providence Health Care) sites. The study also received ethical approval from the University of Toronto, the New York Academy of Medicine and Johns Hopkins University.

The main objective of the NAOMI Study is to determine whether the closely supervised provision of injectable, pharmaceutical-grade heroin is more effective than MMT alone in recruiting, retaining, and benefiting chronic, opioid-dependent, injection drug users (IDUs) who are resistant to current standard treatment options.

The final version of the NAOMI study was a two-site study (Montréal and Vancouver) with 251 participants randomized to receive either injectable opioid or methadone for 12 months of treatment (plus 3 months of transition off of injectable medication), plus 12 months of research follow up. Participants randomized to the injection arm self-injected medications under the supervision of clinic staff and could add oral methadone at any time during their treatment in consultation with their physician. The target population for NAOMI included men and women over the age of 25 who were chronic, opioid dependent, daily IDUs and who had previously failed MMT. Further information on the trial design and methodology are described elsewhere [47].

### NAOMI – science vs. politics

The line between science and politics can be blurred at times. Generally, researchers are most concerned with the best science; however, in addiction research, getting a RCT approved and funded sometimes means that certain compromises must be made. This can prove to be challenging, as was the case with NAOMI, as researchers worked to design a study that would be politically acceptable while at the same time maintaining scientific integrity and ensuring that the protocol would be scientifically rigorous. This issue brought the distinction between pharmacology and phenomenology to the forefront of explaining and educating people on opioid addiction.

The harms of heroin use can be categorized into two classes, pharmacological and phenomenological. Pharmacology refers to the science of drugs, including their composition, uses and effects, while phenomenology incorporates the fact that events and peoples' lives occur in a complex and multi-faceted environment where the socio-political systems affect people's lives. The pharmacological harms associated with heroin use include: euphoria and/or sedation; withdrawal; constipation; and flushing, many of which are also associated with other commonly prescribed opioids. However; the phenomenological harms associated with heroin use, the ones most often discussed, include: overdose; viral infections; bacterial infections, violence, illegal activity; and social disintegration. The latter are not attributable to the pharmacology of the drug but rather to drug prohibition which leads to the use of dirty needles, unsanitary/non-sterile water, crime, prison, disease and overdose. The question that comes to mind is 'What if all these 'additives' were removed from street heroin? What would be left?' The answer is diacetylmorphine or DAM, the active opioid contained in heroin.

Heroin has a documented history of being prescribed and used as an analgesic dating back to at least 1901 when Bayer^® ^Pharmaceutical Products were marketing it. Between 1919 and 1923 there were several morphine and HAT clinics operating in the USA until their termination by the US government. By 1926 UK physicians were officially allowed to prescribe heroin; however, limitations and controls were placed on heroin prescription in 1965 resulting in only limited numbers of patients being currently prescribed heroin in the UK [32].

Due to the controversial nature of the drug being tested in NAOMI, the study design of the protocol was driven in some places, specifically in the design of the eligibility criteria for the study. For example, the study could not recruit participants currently in MMT or who had been in MMT in the prior 6 months because of the 'fear' that some individuals would drop-out of treatment in order to apply for the NAOMI trial. This criterion was an amendment to the original protocol in response to health authorities and MMT providers. In contrast, two of the other trials published on HAT required that participants 'must' be on MMT in order to participate because HAT was projected to become part of the addiction treatment system. As a result, many advocates and drug users felt that the NAOMI study entry criteria were far too restrictive and that, if the intent was to truly help this largely underserved population, the criteria should be more flexible and inclusive.

Another somewhat political NAOMI issue was the withdrawal of the Toronto site from the study. Construction had begun at the Toronto site; however, they experienced various delays in the renovation process. This together with the previous commitment of the Toronto site to run another study in the renovated clinic before they could start NAOMI, meant that there would be a significant time delay between when Toronto could start the trial and when the other two sites would be starting. In addition, there was a lack of consensus amongst the physicians and researchers at the Toronto site as to whether heroin or diverted prescription opioids were really the primary issue in that city. This lack of consensus then extended to the feasibility of the Toronto site being able to fully recruit into NAOMI and what the potential value of HAT would be in the Toronto context. Interestingly, a 2003 paper written by researchers from the Centre for Addiction and Mental Health in Toronto (CAMH) analyzing a population survey conducted amongst adults residing in Ontario regarding public opinion of safe injection facilities and HAT showed that 62.3% of participants questioned felt that medically prescribed heroin should be available for long-term heroin addicts who have tried and failed all other treatment options [48].

### NAOMI in Canada

The NAOMI protocol was submitted to CIHR in March 2001; however, at the time CIHR was not able to provide full funding for the trial. Consequently, CIHR spent several months trying to identify additional sponsors. (The US National Institutes of Health (NIH) were approached but declined to co-sponsor the study.) In January 2002 full funding approval was granted for NAOMI after CIHR received an overall funding increase.

An important point to note about the NAOMI study is that, unlike its counterparts in Europe, NAOMI was created and conducted by private citizens. In contrast, the European HAT studies were government initiated and thus already had the support of government and funding bodies. This distinction is important in understanding many of the hurdles in starting recruitment and treatment into this type of study in North America.

With CIHR approval and funding in hand, a Clinical Trial Application (CTA) needed to be written for submission to the Regulatory Branch of Health Canada. Since there was no pharmaceutical company 'sponsor' for the NAOMI study, the Principal Investigator became the sponsor and thus the study team prepared and submitted the CTA. During this somewhat lengthy process, the first hurdle (aside from funding) appeared. The study team was informed that the bulk powder form of heroin that had been planned to be used for the study was not acceptable to Health Canada due to contamination/sterility concerns. Instead, Health Canada required that the narcotic be imported in lyophilized/freeze-dried form resulting in a cost that was approximately eight times more expensive than originally planned.

Final CTA approval for the study was granted in January 2003, which enabled the team to focus on the next steps of getting the trial up and running, specifically identifying study sites, seeking import/export permits for the heroin/DAM, and obtaining a special section 56 exemption to Canada's *Narcotics Control Act *to allow the team to import, receive, and administer the heroin/DAM without the staff or participants being arrested. This is the same exemption that Vancouver's Supervised Injection Site required/s; however, the requirements to obtain this exemption varied greatly between the NAOMI study and the Supervised Injection Site (also know as Insite).

### Identifying study sites

It was thought that identifying study sites would be a relatively straightforward and uncomplicated task, as was the case with the Montréal study site. However, identifying a Vancouver study site was anything but uncomplicated.

One of the first sites discussed for Vancouver was St. Paul's Hospital, the city's inner-city hospital, which predominantly deals with Vancouver's IDU population. However, at the outset multiple concerns were raised. First, St. Paul's Hospital is a Catholic Hospital and, as such, was fearful (at the time) about being perceived as condoning or supporting injection drug use; Second, there was concern by the hospital that having the study site located within its walls would result in those addicted to heroin moving into the neighbourhood in order to have easier access to the site, known as the 'honey pot effect'; and finally, after further consideration, it was the study team's feeling that having the site at St. Paul's Hospital would be an impediment to participation as the target population would have to travel three times per day to get to the site. Although the hospital is only located 1.25 miles away from the current site in Vancouver's Downtown East Side (DTES), NAOMI's target population is deeply rooted in this community due largely to poverty, housing, welfare, and stigma issues.

Vancouver's DTES is often described as the "poorest urban postal code in Canada" and is home to approximately 5,000 IDUs. The median household income in the DTES in 1996 was $12,900 compared to the City average of approximately $48,000 [49]. According to a March 31, 2008 Expert Advisory Committee report to Canada's Federal Minister of Health, results based on 1,000 users surveyed in the DTES showed that 20% are homeless and many more live in unstable and/or single resident rooms. 80% have been incarcerated at some point, 38% are involved in the sex trade, 59% reported a non-fatal overdose in their lifetime and 51% used heroin as their primary drug of choice. A report prepared by Coroner J.V. Cain (*Report on the Task Force into Illicit Narcotic Overdoses in British Columbia*) [50] reported that overdose deaths in Vancouver had risen from sixteen in 1987 to two-hundred in 1993. In 1997 researchers at the British Columbia Centre for Excellence in HIV/AIDS reported that the prevalence rate of HIV/AIDS in the DTES had reached epidemic proportions and was 27% among injection drug users at the time. In a September 1997 report by the Chief Medical Health Officer of the Vancouver Richmond Health Board it was reported that many individuals in the DTES were coping simultaneously with poverty, lack of affordable housing, lack of transportation, inter-generational abuse and violence, and poor access to services. The majority of users in the DTES are either on welfare, are unemployed, or do not qualify for welfare. These factors make travel to St. Paul's Hospital up to three times per day, seven days per week, 365 days per year, a significant challenge. Based on this and other circumstances, it was decided that the study site should be established in the DTES.

Identifying a final study site in Vancouver took almost two years. During this time many sites were considered. Some were proposed by the local health authority, some proposed by local service organizations, and others found by members of our team walking the streets of the DTES looking for vacant buildings. Unfortunately, the sites initially identified in the DTES all had one major issue in common, they all required large amounts of renovations, often estimated at close to $1 million Cdn worth, just to be able to obtain an occupancy permit. Moreover, this $1 million price tag often did not include the cost of the significant safety and security additions that the government would require in order to obtain the necessary Section 56 exemption to legally store and administer the drug.

The first viable potential site identified was located in a neighbourhood just on the outer edge of the DTES. This site was centrally enough located so that the participants could easily access it, and was also manageable in terms of renovation costs. However, some members of the local community had concerns about having the site for this type of clinical trial in their backyard (the 'Not in My Backyard' or NIMBY issue) and thus petitioned the City against our use of it. In its reasons for not wanting the NAOMI site in their neighbourhood, the community cited that they felt that the overall area already housed many service organizations and, thus, did not need another one. Because of these issues, this site had to be abandoned.

It took nine months from that point to identify, secure, and sign a lease for what is now the site of the Vancouver NAOMI clinic. This site is in the heart of the DTES in a former bank, complete with a vault, thus reducing some of the renovation expenses in terms of the requirements for the Section 56 exemption. In order to proceed with renovations, the Vancouver study team needed to apply to the City of Vancouver for a development permit. At the time, the study team thought that this process would be relatively painless as the site was not in a predominantly residential neighbourhood; however, once again the site became a political issue as some residents had concerns about the site, specifically the NIMBY issue and the number of service organizations already in operation in the DTES. This resulted in our application having to go to a full City Development Board review. The review process resulted in the need for a public City Development Board meeting to be called where both the study team and concerned residents could voice their feelings. Within a few months the meeting was convened and concerns in the community had died down resulting in only two people attending the meeting to speak out against the site location. With the backing of some local service organizations, including the invaluable support of Vancouver's Police Department, approval of our application was granted and renovations could start; however, not without some unique conditions.

### City conditions for the Vancouver site

In response to the community's initial concerns, and given the challenges that the City of Vancouver (and other cities) face in managing the broader environment of facilitating increased services in a neighbourhood that already is perceived by many to have too many services, some specific conditions were attached to the development permit approval of the Vancouver site by the City of Vancouver, including: 1) A twenty-four hour emergency contact telephone number be established and posted on the doors of the site as well as distributed to the community so that, in the event that members of the local community had any concerns, they could contact the study team at any time. It is noteworthy that this line did not receive a single call with a complaint about the study site. 2) The study team had to guarantee that there would not be line-ups out front of the clinic at any time and, should such occur, the team would disperse the line up. This is a demonstration of some of the stigma that affects this already marginalized group, especially given that businesses, such as ticket stores, box offices and electronics store, as well as businesses that serve alcohol such as bars, lounges and night clubs, are generally allowed to have line ups in front of their establishments. 3) The study team had to develop a 'Good Neighbour Agreement' which each participant was required to sign upon receiving their designation (injectable opioid or methadone). This agreement required the participants to agree that they would not loiter near the clinic, not line up outside of the clinic, not arrange to meet people directly outside of the clinic, and not deal any drugs in the area directly surrounding the clinic. 4) The study team had to strike a Neighbourhood Advisory Committee (NAC), to which any interested groups in the area surrounding the clinic (businesses and housing strata-committees) were to be invited to participate. This committee was to meet on a regular basis, with Vancouver study team members in attendance, so that they could voice any concerns about the study and its effect, if any, on the neighbourhood. The NAC started meeting in early 2005 and continued to meet until the clinical portion of the trial completed. During this time the NAC did not raise any concerns regarding the study. As a matter of fact, the NAC describes the study as a 'non-event' in terms of its effects on the community. This is to be expected considering the study is only able to treat 192 of the estimated 5,000 IDUs in the DTES. 5) Due to concerns raised over the 'honey pot effect', specifically those addicted to heroin moving into the already crowded DTES from other areas in order to access the study, the study was restricted to recruiting only those participants who lived within a one-mile radius of the study site. While this restriction certainly limited recruitment to participants that were truly the most visible chronic opioid addicts, it also excluded many people with a heroin addiction who truly did need help, were ready for treatment, and met all of the inclusion criteria except the latter residency restriction. To our knowledge no other clinical trial has had restrictions on recruitment by geographic area placed upon it by the City of Vancouver. All of these conditions speak to the chronic institutionalized discrimination affecting drug users.

It is important to note that the residency criteria for entering the study was relaxed somewhat in the late summer of 2005. The research team and the City of Vancouver agreed to expand the definition of 'residency' to include people who could prove that they were clearly accessing services on a regular basis within the one-mile catchment area. The City required that the NAC approve of this change, which happened in September 2005, before the change could take affect.

Lastly, as part of our development permit, we were required to provide 'elevation drawings' to the storefront and façade of the study site with the stated intent being to 'improve the appearance of the storefronts to ensure pedestrian interest'. This condition is ironic because the City and the Federal Government did not want to draw attention to the site or what was happening within its walls.

### Health Canada requirements

Aside from the aforementioned City requirements, in order to gain the approval of the Office of Controlled Substances of Health Canada and obtain a Section 56 exemption, additional security requirements had to be met at the study sites. As previously mentioned, the Section 56 exemption is the same exemption that Vancouver's Supervised Injection Site is required to have. However, due to the NAOMI study storing, preparing, and administering narcotics many additional security features were required for the study sites.

The first restriction placed upon the study team was one of confidentiality. Health Canada's Office of Controlled Substances was very concerned that news of the NAOMI study would get out to the Canadian public in advance of their final approval and granting of the Section 56 exemption. They did not want news of the NAOMI trial application in the press while it was still under consideration for approval. Due to this restriction, when news of the first site broke and media were informed about the site and our intentions, the study team was not able to comment to the media about the specifics of the trial, nor that it was a scientifically approved RCT with ethical approval at all of the study sites. This inability to consult with the local community played a large part in the loss of the first study site. This issue continued to be problematic until, finally, there was agreement that the need to inform and educate the public about the trial outweighed any possible benefits that could be gained by remaining silent on the issue.

In addition to the confidentiality issue, Health Canada put forward a host of additional security restrictions which needed to be satisfied in order to gain a Section 56 exemption. The level of security required is determined by the maximum street value of heroin to be stored at the facility at any one time based on Health Canada estimates of street value. However, contrary to common knowledge about the street value of heroin (approximately $30 CDN per 0.25 gram on June 13, 2008), Health Canada policy valued heroin at $3 million per kg or approximately $750 per 0.25 gram, roughly twenty-five times more than the actual street value.

This Health Canada policy resulted in the security required for the study sites to soar to a whole new level. The long list of Health Canada requirements included: specially reinforced glass; a sealed medication room with a specially designed one-way tray through which nurses passed medications to participants; a chute in the reverse direction through which participants returned used syringes under observation; multiple security cameras covering every angle, including overlap, to ensure that no heroin was diverted, either by participants or staff; vestibules to be built so that there was a 'secured' area and people could not directly enter the clinic through one door – this would provide the clinic staff with the opportunity to vet, via security camera, those who entered the vestibule before they were actually permitted access to the clinic proper; although the site was only able to keep a three day supply of heroin on the premises due to other Health Canada restrictions, the heroin had to be stored in a locked fridge within a safe to which only the clinic managers had keys and passwords; specific safety training was required for all staff including how to deal with a hostage situation (Health Canada was concerned about members of organized crime wanting to obtain the heroin stored at the clinic); and the development of a specific and detailed system to log and monitor every milligram of heroin from the time of delivery to administration. In addition, every time point had to be recorded for each participant visit including when they enter the premises, when they pass their pre-assessment, when they began and complete administering their dose, whether the patient left any drug visible in the syringe, when they pass their post-assessment, when they are discharged, and any other interaction with study nurses, physicians, social workers, addiction counsellors, medical office assistants, or clinic managers.

As the Vancouver site was not attached to an institution such as a hospital, it had additional requirements placed upon it, including: only a very select few could have keys and passwords to enter the clinic when it was not in operation – clinic staff would have to be granted entry access from a clinic manager already inside the clinic; and vibration sensors had to be installed on all of the outside doors and windows. Given the nature of all of these requirements, there were significant corresponding costs associated with establishing these clinics that could not be covered by the original scientific grant. These additional security requirements, as well as the change in the heroin compound that Health Canada would allow the study team to utilize, resulted in roughly an additional $2 million Cdn needing to be raised in order to establish, recruit into, run, and complete the study. The final funding for the study is derived from several different funding partners, each with their own restrictions on expenses and individual reporting requirements, thus necessitating internal accounting staff dedicated to the management of the NAOMI study budget, including transaction processing and financial reporting. The final budget for the study, including one-time costs such as renovations and security, is approximately $10.5 million Cdn.

The last issue to be resolved with Health Canada was how the heroin would be delivered to the clinics. While it was acceptable to have the heroin delivered to the hosting, institutional pharmacies by regular couriers who routinely deliver medicines, Health Canada was very specific that the study medication be delivered to the study sites by armoured car. In fact, there was a point when the study was not going to proceed despite the work to-date because Health Canada was insisting on daily deliveries of the study drug (meaning that only a one day supply of drug could be stored on-site as opposed to a three day supply). This resulted in lengthy discussions during which the study team outlined to Health Canada that should a strike occur (at the armoured car company), or should weather impede the daily delivery of study medication (which was very likely at some point in Montréal), then the study team would not be able to provide the medication as outlined in the protocol. Further, this would be considered a breach of ethics as well as a breach of Good Clinical Practice (especially since such a delay could be foreseen and precautions taken against it).

Finally, a compromise was reached. Health Canada and the researchers were able to come to an agreement on the amount of heroin that could be stored at the clinical sites at any one time, thus no longer necessitating daily armoured car deliveries.

### Other challenges – our neighbours to the south

One of the other challenges is the proximity and close relationship of Canada and the US. While there are many supporters of harm reduction policies and practices in the US, the Bush administration is not among them and is well known for its lack of support for important harm reduction programs such as needle exchange programs, as well other addiction treatment initiatives. When it comes to clinical trials providing heroin for treatment of heroin addiction or Supervised Injection Sites, there is no question about how the Bush administration feels about such interventions. In 2003 US Drug Czar John Walters referred to Vancouver's Supervised Injection Site as "state sponsored personal suicide" [51], and in interviews on Canadian television made what some would call veiled threats saying that it would be "regrettable" if Canada started having trouble getting their goods across the (US/Canada) border [52].

While the general expectation is that politics should not influence science and research, the fact is that politics can play a significant part. Aside from the political issues earlier identified with respect to the eligibility criteria for the study, there were, and continue to be, politics around areas of harm reduction. In the Canadian context, the current Conservative Federal Government has been working to strengthen ties with the US Government and is often unwilling to confront the US government, especially for a cause as controversial as HAT.

In 2004 the NAOMI team in Vancouver received a request from the US Consulate in Vancouver for a meeting. US Embassy officials visited the Principal Investigator and a member of the NAOMI study team and, while the first part of the conversation was centred around the study itself, the officials also requested (and were denied) a list of our US collaborators, specifically the US members of the NAOMI Working Groups established in 1998.

### Recruitment into NAOMI

In March 2005 recruitment into NAOMI commenced in both cities. A lot of time and energy was spent on developing recruitment strategies. Due to the nature of the study, and also because the study had been receiving media attention prior to the start of recruitment, the study team expected a deluge of calls once the recruitment lines were open. With only a relatively small amount of research assistants available due to budget constraints, the phone lines were initially open two days per weeks for two hours at a time. Posters describing the basic entry criteria into NAOMI, the call in telephone numbers, and the hours when the phones would be manned, were put up around the DTES and in the City of Montréal, at needle exchanges, community services and at VANDU (the Vancouver Network of Drug Users). However, the expected deluge of calls did not materialize. Some of the factors leading to this were that: 1) many of the people NAOMI was trying to reach were homeless; 2) limited access to telephones during the few hours the lines were open; 3) the gag order imposed by Health Canada before the study began had limited general education about the NAOMI study; and 3) some doctors and community services/organizations were incorrectly informing potential participants that the study was already full. In response to this, a new recruitment strategy needed to be developed quickly.

The first change was to open up the recruitment lines during normal business hours. The study teams also designed new posters, created a schedule to regularly go out into the community and replace torn posters, and hired outreach workers. These outreach workers were charged with going into the community and talking with users. They visited needle exchanges, community centres, local organizations, and the Supervised Injection Site (in Vancouver). Outreach workers would conduct a 'pre-screening' questionnaire to determine if a person might potentially qualify for the study, and would then book an appointment or even accompany the participant to the research office where full eligibility could be firmly determined.

In addition to the outreach workers, team members conducted information sessions for community groups and services, as well as for groups of doctors, nurses and other workers in the communities and in other addiction arenas. In order to reach the population that the NAOMI study was seeking, advertisements were placed in the local 'free' papers, team members wore NAOMI t-shirts when they were out in the community, recruitment and information sessions were held where users were known to congregate, and matchbooks were created and distributed which listed the basic entry criteria into the study and the telephone call-in numbers.

Not surprisingly, recruitment into the study took longer than expected, finishing in April 2007. However, the main impediment to recruitment was not the factors listed above, but rather the highly restrictive nature of the inclusion/exclusion criteria. It is noteworthy that delays in recruitment have been a barrier in most of the trials aimed at hard-to-reach drug using populations [53,54]. For example, the German HAT RCT had to be extended an additional year due to unexpected delays in recruitment into the study [55]. In the case of NAOMI, the primary barriers to study entry were in relation to previous addiction treatment attempts and of not being on MMT at the time of recruitment. The research assistants found that many potential candidates had not reached a dose of 60 mg of methadone and/or had not remained on methadone for at least one month in their previous attempt(s). Many individuals currently or recently on MMT but not doing well came forward but were generally ineligible because only those without any addiction treatment in the last 6 months were able to participate. Other issues with less impact were: not having resided for at least one year in the city/site location, and for Vancouver participants, not living within 1 mile of the clinic, not meeting the (later) relaxed criteria of being a resident of the DTES, or self-report of living within the one mile radius but not able to obtain any documentation to verify the claim.

In Montréal, some of the issues surrounding recruitment differed from the Vancouver site. Unlike Vancouver, Montréal's heroin users are spread over the island and there is no large concentration of heroin addicts in one area comparable to the DTES. Also, without a central provincial prescription database like Pharmanet in British Columbia, obtaining the required verification of previous MMT treatments for potential Montréal participants was difficult. Further, because Montréal's IDU population is fairly spread out, many would have to travel up to an hour to the clinic up to 3 times per day, which was not seen as very attractive even with the provision of 'free' heroin.

At the end of the recruitment phase, the profile of the participants who entered the NAOMI trial represented some of the most chronic and marginalized opiate users in Vancouver and Montréal [56].

### Working with the media

Another challenge relating to NAOMI has been media attention. Since publicly announcing the start of the trial, the study has received considerable attention in local, national and international media. While media interest can be beneficial in raising the profile of harm reduction, the media's need for headlines can throw off the balance between scientific integrity and public education.

Treating heroin addiction with heroin tends to evoke a knee-jerk reaction. Lack of understanding, restrictions on time and resources, and the need for a catchy headline often lead to sensationalism by the media. As previously mentioned, opposition both within Canada and the US also contributed to misleading reports from local, national, and international media. The resulting focus has been on a seeming shift in Canadian drug policy in direct contradiction to the US war on drugs, rather than on the scientific or medical merits of the NAOMI study.

With the NAOMI study being the only study of its kind in North America, and with its two study sites located within close proximity to the Canada/US border, increased media attention was unavoidable. Despite the fact that the NAOMI study targets a very specific and marginalized group within restricted geographic locations, regular bursts in media attention have required the team to extend strategic communications and outreach to a much broader audience.

Some of issues raised in opposition to NAOMI are similar to those raised for HAT in general. Many of them are based on fear and misinformation, while others are based on different moral values. Like many new treatment modalities, critics exist who are opposed to NAOMI as well as HAT. These criticisms are seen as improvement opportunities for those who support HAT. A lot has been said about this issue, and the authors refer the readers to the early papers that discuss this topic in depth [57-59].

One myth about HAT is that it is a better treatment then MMT and thus would replace MMT. In fact, HAT is not meant to replace MMT, but to be another available treatment option. One that would have a small but very important role in the addiction treatment system [46]. Even in two of the three countries where HAT exists as a regular program, participants in the program account for less than 10% of those in substitution treatment in those countries [31,45]. Another common myth is that HAT patients will start using more cocaine (and/or other drugs) because they no longer have to purchase heroin. The results of all the RCTs showed that the use of cocaine remains stable among HAT patients or even declines [37,40,55,60].

Also an issue for some of the critics of HAT is that heroin is a respiratory depressant and daily injection is less safe than non-administration [40,41]. While injecting three times per day, or even daily, is clearly more harmful than not injecting, the analysis of patient safety should take into account that if the patient is not receiving HAT he/she would likely be injecting street heroin (cut with other additives) in an unsafe environment, not to mention the illegal activity that many enter into in order to purchase their drug(s). This rationale is supported by the Swiss study where in a seven-year period the mortality rate of the Swiss participants was 1% per year, which is very low compared to the general mortality ratio of Swiss opioid users (2.5–3%) [61].

Further complicating the issue, North America's only Supervised Injection Site (Insite) operates only blocks away from NAOMI's Vancouver site. This had lead to confusion amongst media and in turn the public who may not fully understand the difference between NAOMI (a randomized controlled clinical trial) and the Supervised Injection Site (an important local harm reduction initiative).

Science and media work in different ways and have different priorities. Bridging this communications gap can prove to be challenging and frustrating. The NAOMI team addressed this issue through a concerted effort to educate local communities, to maintain a low profile nationally, and to commit to scientifically based messaging.

Due to these evolving issues the need to bring a Communications consultant on board was identified in order to help define a response and to respond to media. This person was charged with putting together the text for a study website , writing press releases, responding to basic media queries and arranging interviews which became a huge part of the overall NAOMI study. While some journalists presented thoughtful and engaging perspectives on heroin therapy, incomplete and intended-to-shock media reports, not only within Canada but also internationally, continued to stir emotions and increase anxiety among local communities, police, various levels of government, and our neighbours to the south. Having someone on the team to help manage and respond to these types of issues was very beneficial.

The NAOMI team's media strategy continues to be to respond to media whilst committing to an unwavering focus on the scientific aspects of the study. As discussed earlier, the team also conducted community consultation meetings and made numerous presentations to local services organizations such as the Vancouver Area Network of Drug Users (VANDU), and to local community organizations, the Provincial Government and the Federal Government. In addition, Community Advisory Boards/Committees (CABs) were established in Vancouver and Montréal. The Vancouver CAB included representatives of local community groups such as Grief to Action and VANDU, the Provincial Health Officer, and also representatives from the Royal Canadian Mounted Police, Vancouver Police Department, British Columbia Ministries of Health and Attorney General, British Columbia Centre for Disease Control, and the City of Vancouver. The Montréal CAB had a similar make-up comprising of representatives from municipal, provincial and federal governments and police, health authorities, pharmacists and physicians, public security, ex-injection drug users, as well as representatives from addiction treatment centres and the Université de Montréal.

Over time, headlines and attitudes shifted from "Clinical disorder on our northern border" (The Hoya, Georgetown University) to "Why it makes sense to give them heroin" (The Globe & Mail, Canada). More recently, the Vancouver Courier published an article on NAOMI entitled "End of NAOMI wastes research" (April 2, 2008) describing that the study was due to finish this year and the chances of HAT being turned into a government supported program were unlikely.

### NAOMI – status report

The last of NAOMI's 251 participants completed the clinical portion of the study in June 2008. While the Montréal clinic is now closed, part of the Vancouver clinic remains open and is now functioning as a small MMT clinic.

At the end of the 12-month period of active treatment, participants were transitioned to available therapies in the community, primarily MMT. Thus, even those who were responding to HAT had to stop this treatment and go back to the same options that had not worked for them in the past because the study team could not legally continue to prescribe DAM outside of the clinical trial treatment period. Retention in treatment at the 12-month point was significantly higher in the injection arm than the oral arm. Results of the primary outcomes were released publicly on October 17, 2008 and are available on the study website at  (scientific publication pending).

Compassionate access to DAM for a small sample of NAOMI participants who were retained, responding, and had benefited from HAT, was sought through Health Canada's Special Access Programme. However, the requests were denied as the Programme felt that 'there are other options (i.e. marketed drugs) that we would consider alternatives to diamorphine at this time'. The results of the primary end points demonstrate that, like in other countries, HAT can attract, retain, and benefit this sub-population. However, as the Federal Government, through Health Canada, has denied compassionate access use to DAM, HAT treatment is unavailable in Canada. Canada is the only country where diamorphine has been tested for addiction treatment and has been denied compassionate use [62].

On a separate but related issue, recently the Supreme Court of British Columbia ruled that Section 4(1) and 5(1) of the Canadian *Controlled Drug and Substances Act *(CDSA) are inconsistent with section 7 of the Canadian Charter of Rights, thus meaning that those sections of the CDSA are of no force or effect. This translates to Vancouver's Supervised Injection Site being able to remain open despite Federal Government. Specifically, the judgement grants "users and staff at the Supervised Injection Site, acting in conformity with the operating protocol now in effect, a constitutional exemption form the application of ss. 4(1) and 5(1) of the CDSA." However, the Canadian Federal Government is appealing this ruling [63].

While not all of the 18 month and 24 month follow-up research interviews have been conducted as of yet, and the results of these interviews are not expected to be disseminated until summer 2009, it appears that after the treatment endpoint participants generally either have returned, or are returning to, where they were at baseline. In a few cases the treatment participants accessed through the study acted as a springboard to more long-term stability. However, it appears that a much larger proportion of participants have returned, or are returning to, where they were at when they first entered the study.

As previously mentioned, the Vancouver clinic is partially open and is providing MMT to some NAOMI participants, as well as other patients from the community. The study team hopes that the clinic will also be prescribing hydromorphone in the future. While details have not been finalized, and funding remains an issue that needs to be resolved, the team is hopeful that this opportunity will pan out. However, the possibility of providing heroin treatment seems less likely given the conservative nature of those that would need to provide approval and given the response to-date from Health Canada's Special Access Program. Notwithstanding, the NAOMI team continues to work to try and make access to heroin and/or hydromorphone available to NAOMI participants and hopefully others who are in need of, and want, treatment, and who would be good candidates for a form of HAT therapy.

## Conclusion

Despite the challenges surrounding NAOMI, the authors firmly believe that the study was worth doing. Given the political issues still surrounding HAT, the authors feel that it was important to conduct a HAT RCT in Canada and look at HAT in the Canadian context. Although HAT is not approved it Canada at this time, the authors hope that this will change one day given the NAOMI results, in combination with the other HAT trial results already published.

If any of the authors, or anyone else associated with the study, were to ever doubt whether NAOMI was worth doing, or the profound affect it had on some participants, we need only to remember the words recently written to the Principal Investigator of NAOMI by one of the NAOMI participants:

*"...I want to tell you what being a participant in this study did for me. Initially it meant "free heroin". But over time it became more, much more. NAOMI took much of the stress out of my life and allowed me to think more clearly about my life and future. It exposed me to new ideas, people (staff and clients) that in my street life (read: stressful existence) there was no time for*.

After NAOMI, I was offered oral methadone, which I refused. After going quickly downhill, I ended up hopeless and homeless. I went into detox in April 2007, abstained from using for two months, then relapsed. In July 2008 I again went to detox and I am presently in a treatment center...

I am definitely not "out of the woods" yet, but I feel I am on the right path. And this path started for me at the corner of Abbott and Hastings in Vancouver...

Thank you and all who were involved in making NAOMI happen. Without NAOMI, I wouldn't be where I am today. I am sure I would be in a much worse place."

## Abbreviations

All abbreviations used in the text were defined in the text where first used.

## Competing interests

The authors declare that they have no competing interests.

## Authors' contributions

CCG has been involved with the study since late 2002. The case study submitted is an account of events prior to 2002, and the majority of the case study focuses on events after 2002, which CCG was directly involved with. CCG drafted the article, coordinated the study, chronicled the events, and researched many of the references.

EOJ researched the references listed in the case study and added intellectual content for the case study.

NL was also involved with the study in the early days and added intellectual content for the case study.

MTS is the principal investigator of the study, was involved in revising the case study, and gave final approval of the version submitted.

## Authors' information

CCG is the National Coordinator for the Study. CCG is also the Chief Operating Officer for the CIHR Canadian HIV Trials Network. EOJ is a researcher and analyst for the Study. She was also a lead investigator in the Spanish HAT Trial. NL became involved with the study shortly after it was approved by CIHR and was the Vancouver Clinic Coordinator for the study. MTS was an initial member of the first working group and is the Principal Investigator of the Study.

## Consent

Written informed consent was obtained from all persons who participated in this study. A copy of the written consent in available for review by the Editor-in-Chief of this journal.
